# Textile Pressure Mapping Sensor for Emotional Touch Detection in Human-Robot Interaction

**DOI:** 10.3390/s17112585

**Published:** 2017-11-09

**Authors:** Bo Zhou, Carlos Andres Velez Altamirano, Heber Cruz Zurian, Seyed Reza Atefi, Erik Billing, Fernando Seoane Martinez, Paul Lukowicz

**Affiliations:** 1 German Research Center for Artificial Intelligence, 67663 Kaiserslautern, Germany; paul.lukowicz@dfki.de; 2 Department Computer Science, University of Kaiserslautern, 67663 Kaiserslautern, Germany; carlos_andres.velez_altamirano@dfki.uni-kl.de (C.A.V.A.); heber.cruz_zurian@dfki.de (H.C.Z.); 3 Swedish School of Textiles, University of Borås, 50190 Borås, Sweden; seyedreza.atefi@hb.se (S.R.A.); fernando.seoane_martinez@hb.se (F.S.M.); 4 School of Informatics, University of Skövde, 54128 Skövde, Sweden; erik.billing@his.se; 5 Institute for Clinical Science, Intervention and Technology, Karolinska Institutet, 17177 Stockholm, Sweden; 6 Department Biomedical Engineering, Karolinska University Hospital, 14186 Stockholm, Sweden

**Keywords:** tactile sensing, smart textiles, human-robot interaction

## Abstract

In this paper, we developed a fully textile sensing fabric for tactile touch sensing as the robot skin to detect human-robot interactions. The sensor covers a 20-by-20 cm${}^{2}$ area with 400 sensitive points and samples at 50 Hz per point. We defined seven gestures which are inspired by the social and emotional interactions of typical people to people or pet scenarios. We conducted two groups of mutually blinded experiments, involving 29 participants in total. The data processing algorithm first reduces the spatial complexity to frame descriptors, and temporal features are calculated through basic statistical representations and wavelet analysis. Various classifiers are evaluated and the feature calculation algorithms are analyzed in details to determine each stage and segments’ contribution. The best performing feature-classifier combination can recognize the gestures with a 93.3% accuracy from a known group of participants, and 89.1% from strangers.

## 1. Introduction

Tactile sensors are an important sensory in robotics, since they contribute largely as the synthetic counterpart of biological skins on human and other animals. They are crucial in providing control feedback for safely and securely grasping and manipulating objects [1,2,3].

However, a majority portion of biological skins are not sensitive enough for precise force sensing and localization for assisting controls [4]. In the nature, bodily contact is an important aspect of emotional communication among humans [5], as well as between human and animals [6]. Tactile interaction has been shown to carry emotional information in a similar way as e.g., face expressions [7]. Studies have also shown that body movements are specific for certain emotions [8]. As pointed out in [3], most tactile sensors are based on piezoresistive materials, which have the problem of poor hysteresis and linearity. However, for sensing emotional touches, precision tactile sensing is not necessary [5,7].

In recent years, the focus on robotics research has evolved from precise and delicate movements to perform various tasks, to a deeper communication between human and robotic interactions [9,10,11]. In [12], a humanoid robot WE-4RII (Waseda University, Tokyo, Japan) can effectively express seven emotion patterns with body languages. Touch is fundamental in human-human interaction and as robots enter human domains, touch becomes increasingly important also in human-robot interaction (HRI). In recent years, we’ve seen several approaches to whole-body tactile sensing for robots, e.g., for the iCub (Italian Institute of Technology, Genoa, Italy) [13,14] or the HPR-2 (Kawada Industries, Tokyo, Japan) [15] robots. These systems are cell based, where each cell comprise a small circuit board holding necessary sensors and electronics and, while presenting excellent sensing capacity, they constitute a relatively hard surface with limited flexibility. In [16] conductive serpentine structures and various silicon compounds are used to construct a skin-like bio-compatible sensor to detect touch on different zones and caress across zones.

In a recent study comprising 64 participants communicating emotions to an Aldebaran Nao robot (SoftBank Robotics, Tokyo, Japan) using touch, people interacted for longer time when the robot was dressed in a textile suite [17], compared to a standard Nao with a hard plastic surface. These results indicate that the surface material of the robot may be significant for extending and directing tactile HRI. Inspired by these results, we here report on-going work investigating the use of touch sensitive smart textiles as a potential alternative to cell based robot skins.

One informative aspect of tactile HRI is the type of touch [9]. In [18], individually designed 56 capacitive sensors are installed in a toy bear to detect affection-related touch. The data processing algorithm relies on signal features such as amplitude and base frequency from all the sensors. In [19], a touch sensing furry toy is developed with a conductive fur touch sensor and piezoresistive touch-localization sensor are combined. Using statistical features of the signal and random forests classifier, the prototype recognizes 8 gestures with a 86% accuracy. In [20], using electrical impedance tomography (EIT), with 19 specially placed electrodes, an EIT sensor is used to detect 8 affective touch gestures, with accuracy of 90% for single person, and 71% for multiple participants.

In [21], a commercially available 8-by-8 textile pressure sensor is used. With custom picked features, classification accuracy of 14 gestures is 60% for leave-one-participant-out cross validation. In the present work, we evaluate the capacity of distinguishing between seven different types of touch, listed in Table 1, based on sensor data gathered from the smart fabric.

### 1.1. Contribution

In this paper, we propose to use textile pressure mapping sensors as tactile sensors for emotional related touch gesture detection. We use the smart fabric sensors from our previous research efforts. They have been used for wearable and ambient planar pressure sensing for activity recognition applications, such as monitoring muscle activity through tight fitted textile sensor [22] or recognizing postures from the body pressure profile on the back of office chairs [23].

We define 7 gestures to detect, and evaluate the method through two groups of mutually blinded experiments. The experiment datasets are evaluated by extracting basic statistic features and wavelet analysis. With various classifier, the recognition rate on an exclusive person independent basis is up to 93.3%.

We also analyzed the contribution of each aspect of the feature extraction process, which offers a detailed breakdown of the algorithm and the guideline for reducing the computational complexity with less recognition performance reduction.

### 1.2. Paper Structure

In Section 2, we introduce the fabric tactile sensor and the driving electronics. Section 3 describes the experiment set up and dataset composition; In Section 4, we first explain how to extract features from the spatial-temporal data, then evaluate different features’ contribution to the recognition rate.

In Section 5, we show the detailed classification results as confusion matrix and discuss the results such as the underlying meanings of the miss classifications.

Section 6 concludes the paper with the summary of our findings.

## 2. Sensor Hardware

### 2.1. Sensing Fabrics

The touch sensitive textile skin is made of the textile pressure mapping (TPM) sensor (developed during the EU FT7 project SimpleSkin [24]), which has a three-layer structure as typical force sensitive resistors. But instead of thin film, it is made from flexible fabrics (produced by Sefar AG [25]). Its flexible nature have proven to be suitable for wearable garments, such as a smart shirt [26]; or as textile covers in smart living environments, such as a couch cover for gesture input [27] and posture recognition [23]. Therefore, they can be bent to fit many irregular surfaces as on robots. Compare to other modular, comfort oriented robot skin sensor designs such as [13,14,15,16,28], the fabric TPM sensor has a much simpler structure and less assembling effort. And in contrast to structurally simpler tomography-based sensors such as [29], the TPM sensor offers finer and more reliable pressure location information.

**Figure 1:**
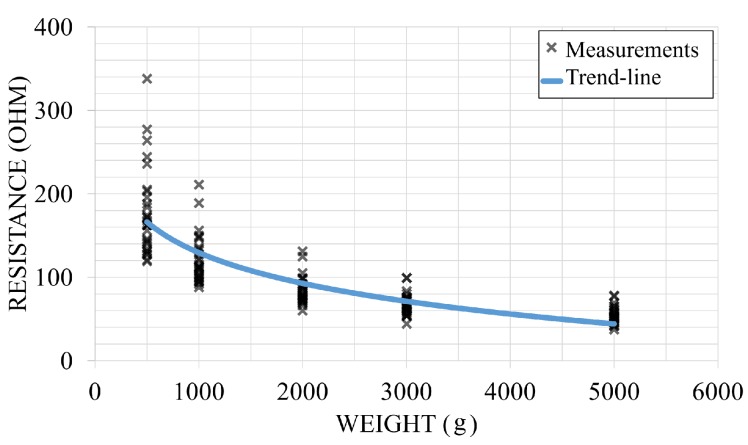
Fabric sensor characteristic.

The middle force-sensitive layer is carbonated polymer fabric, and the top and bottom layers are metallic fiber stripes woven into a non-conductive fabric substrate as parallel electrodes. Every cross point of the top and bottom layers is a pressure sensitive point, and the spatial granularity is determined by the pitch distance of the parallel electrodes. The sensor’s resistance change with the pressed force is shown in Figure 1. A probe is pressed on a random cross point, and multiple levels of force are applied. In this prototype we use a 1 cm pitch, 20-by-20 matrix.

### 2.2. Driving Electronics

**Figure 2:**
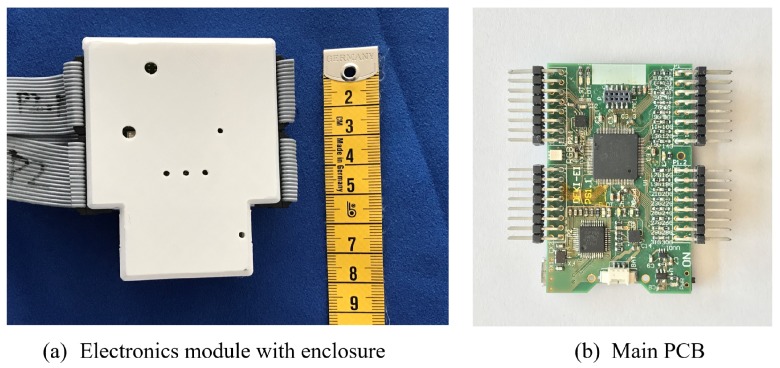
Electronic Hardware.

The sensor is scanned and sampled by a custom designed printed circuit board (PCB) built around a dsPIC micro-controller (Microchip Technology, Chandler, AZ, USA), sampling rate is 50 frames per second with Bluetooth data streaming. It is the same hardware platform we used during our work as a smart chair cover in [23] as shown in Figure 2. The electronic module operates on either wireless or wired for data transmission. With 3.3 V power supply, the sensing part of the electronics consumes 60 mA during operation, and the Bluetooth consumes 40 mA for data streaming. The sensor fabric are connected to the electronic through standard ribbon cables and 2.54-pitch pin connectors, making it easy to produce. On the smart fabric side, the ribbon cables are soldered onto the metallic fabric stripes.

**Figure 3:**
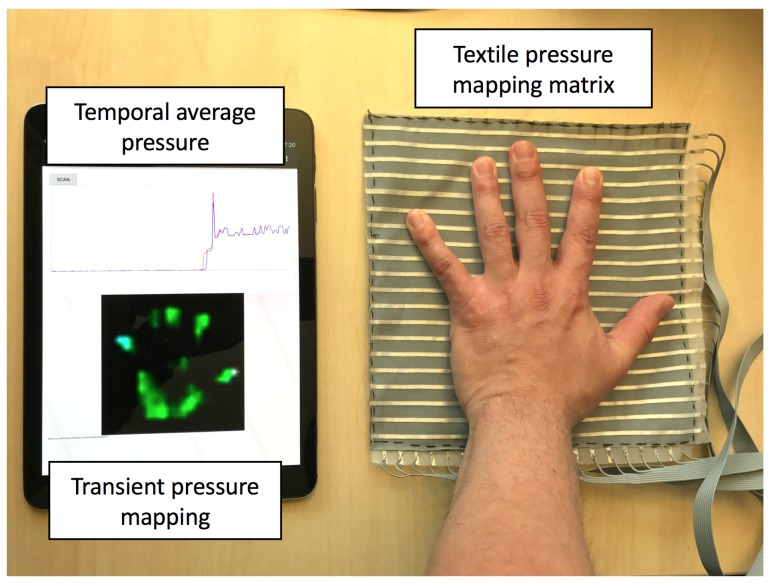
Realtime data visualization with a hand pressed on the textile pressure mapping matrix.

Figure 3 shows the TPM matrix and the real-time data visualizing on a tablet, with a hand pressed in the center. During the construction of the sensor fabric structure, the top and bottom layers of electrode stripes are treated identically; however, on the electronic side, one layer is connected to output ports to provide voltage (stimulus electrodes), and the other layer is connected to analog inputs to measure the voltages (sensing electrodes).

The micro-controller has sufficient pins for both stimulus and sensing electrodes, up to 32 each. During operation, one stimulus electrode is powered with 3.3 V voltage and the remaining stimulus electrodes are connected to ground. The volume resistance $Rsense_{\left(x,y\right)}$ of the carbonated polymer fabric within the overlapping area of one stimulus electrode (indexed as *x*) and one sensing electrode (indexed as *y*) decreases as the fabric is being pressed. Each sensing electrode is connected to a grounding resistor $Rgnd_{y}$. Ideally, $Rsense_{\left(x,y\right)}$ and $Rgnd_{y}$ forms a voltage divider, and the 12-bit analog-digital converter measures the voltage between them, which changes with $Rsense_{\left(x,y\right)}$. A full scan is completed by measuring all of the sensing electrodes when every stimulus electrode is powered. As shown in Figure 3, the hand print is reconstructed on the receiver device by the scanning process.

## 3. Experiment Setup

### 3.1. Experiment Design

**Figure 4:**
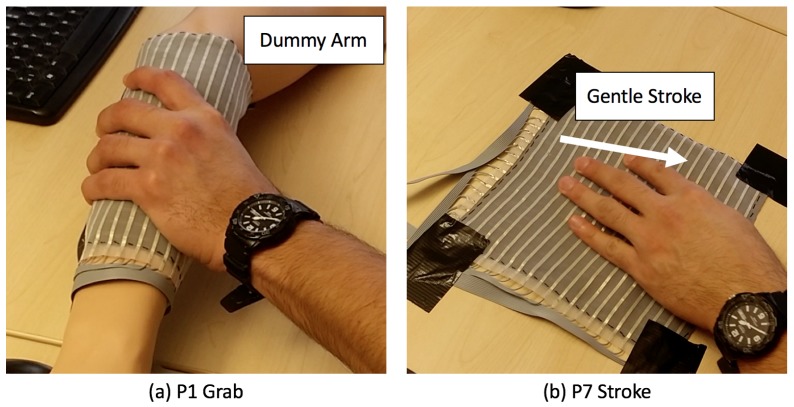
Experiment setup and gesture example (**a**) P1 Grab and (**b**) P7 Stroke.

We defined seven gestures as listed in Table 1. Only in P1 - grab, the sensing skin is wrapped around a dummy arm (Figure 4a), and for the rest of the gestures, the sensor is fixed on a flat surface (Figure 4b). This setup should be seen as a pre-stage to mounting the fabric as skin for a robot, constituting a robot-agnostic baseline without the full complexity of uneven surfaces and the robot’s own motions. The focus of our evaluation is to investigate if the sensor can be used to physically distinguish these expressive gestures.

### 3.2. Dataset

We recorded two groups of data using the same sensor set up. The two groups are conducted by different persons, and they do not have knowledge of how the other group recorded the experiment. The two groups are therefore mutually blinded, including the experiment conductors. It ends up that in Group A, the conductors instructed the participants to perform *P5 Scratch* and *P7 Stroke* as quick burst of three repetitions in a single action; while in Group B, the participants only perform the gestures continuously for every action.

- Group A: twenty four people, two recordings per person. Every gesture is repeated 16 times. During the recording, the participants are asked to use both their right and left hands to perform the gestures equally in multiple repetitions. The participant pool consists of 12 males and 12 females. We assume the hand size may be a contributing influence factor in this experiment. The hand sizes (from the bottom of the palm to the tip of the longest finger) of the males range from 17.5 to 20 cm, and 17–18.7 cm for the female participants. There are one left handed participant in each gender.
- Group B: 5 people, single recording per person. Every gesture is repeated 16 times. The participants use only one hand of their choice to perform all the gestures. There are 2 female and 3 male participants. Their hand size ranges from 16.5 cm to 21.5 cm.

We use Group A for the majority of data analysis and algorithm evaluation. Group B serves as a reference to see how the algorithm is influenced by a completely new setup with different environment and directing persons.

The participants are given the literal descriptions of the seven gestures only, without visual guidance. They are instructed that they can relate each gesture to suitable emotions, however, the experiment instruction does not prescribe specific emotion-gesture bindings. The data is manually annotated to separate every gesture action by the experiment conductor. Overall, 5376 gesture actions are recorded in Group A and 560 in Group B.

## 4. Data Processing Algorithm

### 4.1. Data Format and Digital Processing

**Figure 5:**
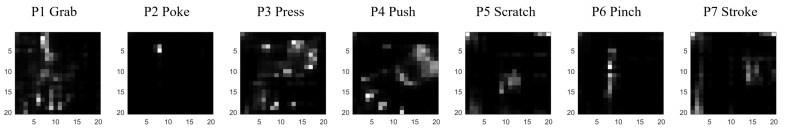
Comparison of the accumulated frame of a random example of every gestures from a random participant. Every pixel in the figure has the summary value of that pixel location within the time window of the action.

The pressure mapping sensor generates a temporal sequence of 2-dimensional pressure mapping *frames*, at a speed of 50 frames per second. Figure 5 shows the accumulated frame of a random example of every gestures from a random participant. Every *frame* is up-sampled from 20-by-20 to 40-by-40 to increase the spatial resolution with bicubic interpolation. To extract information, we first reduce the 2D spatial data into limited information as *frame descriptors*. The following descriptors are calculated from every frame:

- $D_{1}$: mean value of all pixels’ value
- $D_{2}$: maximum value of all pixels’ value
- $D_{3}$: standard deviation of all pixels’ value
- $D_{4}$: distance from center of gravity to the frame center
- $D_{5}$: distance from maximum point to the frame center
- $D_{6}$: the number of pixels that has higher value than a threshold (threshold=mean+standarddeviation)

The frame descriptors therefore reduces the 2-dimensional information to a limited vector. For example, if a gesture lasts 3 s, a stream of 150 frames (each 20-by-20) are generated by the tactile sensor, and six arrays, each 150 in length, are abstracted as the temporal sequences of frame descriptors. $D_{1}$ and $D_{4}$ describes the intensity and location of the center of the pressure; $D_{2}$ and $D_{5}$ offers information of the highest pressure point; $D_{3}$ describes how scattered the pressure is on the surface; $D_{6}$ describes the surface area of the contact.

The experiment data is manually segmented by the experiment conductor roughly before and after the contact. To make sure the data samples cover exactly the contact time, we define a cut-off threshold:

$$Threshold_{cutoff}=min\left(D_{1}\right)+\left(max\left(D_{1}\right)-min\left(D_{1}\right)\right)\times 10\%$$

The samples before the first $t_{1}$ when $D_{1}\left(t_{1}\right)>Threshold_{cutoff}$, and after the last $t_{2}$ when $D_{1}\left(t_{2}\right)>Threshold_{cutoff}$ are removed.

**Figure 6:**
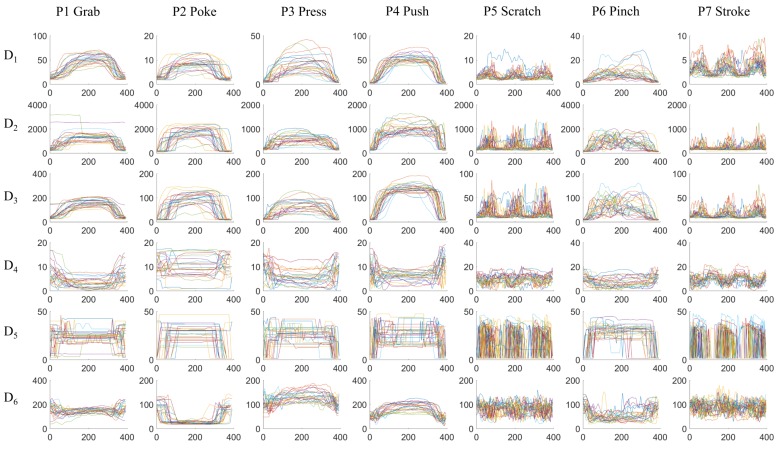
Comparison of the temporal sequence of frame descriptors from each class. Each curve is from a different person within Group A. The x-axis are sample in the temporal domain, the y-axis are scales of the values from different frame descriptors. The original data has different temporal length; in this plot they are scaled to the same 400-sample window using linear interpolation for visual comparison.

Figure 6 visualizes the temporal sequences of *frame descriptors* from different classes. One action of each gesture from every person is randomly selected. For comparison purposes, the sequences are scaled to the same 400-sample window using linear interpolation; the original data sequences have different length. The next step is to extract features to distinguish between different classes. For example, in subplot $D_{6}$-P2, *P2 Poke* has significantly smaller $D_{6}$ than the other gestures; *P5 Scratch* and *P7 Stroke* have distinct higher frequency movements than the other gestures in all the frame descriptors; *P1 Grab* and *P4 Push* has higher average value in $D_{1}$ and $D_{2}$ than the other gestures.

We investigate two types of features from the temporal sequences $D_{x}\left(t\right)$ of size *T*: basic statistic representations, and wavelet analysis.

#### 4.1.1. Basic Features

The 5 basic statistic representations are:

- the average value in the window
  $$mean\left(D_{x}\right)=\sum_{t=1}^{T}D_{x}\left(t\right)$$
- the standard deviation in the window, defined as
  $$std\left(D_{x}\right)=\sqrt{\frac{1}{T-1}\sum_{t=1}^{T}{\left|D_{x}\left(t\right)-mean\left(D_{x}\right)\right|}^{2}}$$
- the absolute range of the sequence: $max\left(D_{x}\right)-min\left(D_{x}\right)$
- the kurtosis of the sequence, which measures how outlier-prone the data is defined as:
  $$kur\left(D_{x}\right)=\frac{\frac{1}{T}\sum_{t=1}^{T}{\left(D_{x}\left(t\right)-mean\left(D_{x}\right)\right)}^{4}}{{\left(std\left(D_{x}\right)\right)}^{4}}$$
- the skewness of the sequence, which is the asymmetry measurement of the data around the mean value. It is defined as:
  $$skw\left(D_{x}\right)=\frac{\frac{1}{T}\sum_{t=1}^{T}{\left(D_{x}\left(t\right)-mean\left(D_{x}\right)\right)}^{3}}{{\left(std\left(D_{x}\right)\right)}^{3}}$$

These features would describe the distribution of the temporal sequence, and are commonly used in statistic analysis.

The temporal features are not to be confused with the frame descriptors. For example, $D_{3}\left(t\right)$ is the standard deviation of all the pixels from a frame at sample *t* at a particular point in time; $D_{3}$ is the sequence of the standard deviation of each frame within the window; $std(D_{3})$ is the standard deviation of all the 2D standard deviation frame descriptors within the window. Frame descriptors reduce the spatial domain data into limited measures, and the temporal features further reduce the temporal domain information. For one window of gesture, 6 sequences of frame descriptors are calculated, which results in overall 30 basic features.

#### 4.1.2. Wavelet Features

To convert frequency-related information from the data into features, we use wavelet transform. Wavelet transform offers frequency and temporal localization of the target signal.

We first pad the $D_{x}\left(t\right)$ signal of length *T* with its boundary values with a padding size of T/2: before $D_{x}$, $D_{x}\left(1\right)$ are inserted T/2 times repeatedly, and at the end of $D_{x}$, $D_{x}\left(T\right)$ are inserted T/2 times. Then the padded signal $D_{x}^{\prime }\left(t\right),t\in \left[1,T\right]$ is multiplied with a symmetric Hamming window w(t),t∈[1,T]:

$$D_{x}^{\prime \prime }\left(t\right)=D_{x}^{\prime }\left(t\right)\times w\left(t\right),$$

$$w\left(t\right)=0.54-0.46cos\left(\frac{2\pi t}{T-1}\right),t\in \left[1,T\right]$$

**Figure 7:**
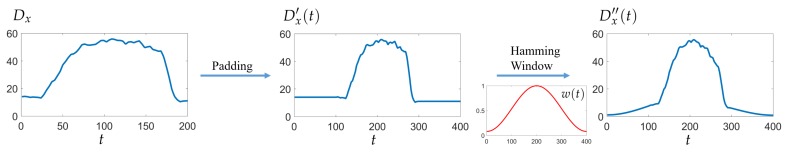
Visualization of the boundary padding and hamming window pre-processing before wavelet transform.

Figure 7 visualizes the boundary padding and hamming window process. Padding and window functions are typical techniques in signal processing to remove the influence of the sampling window.

**Figure 8:**
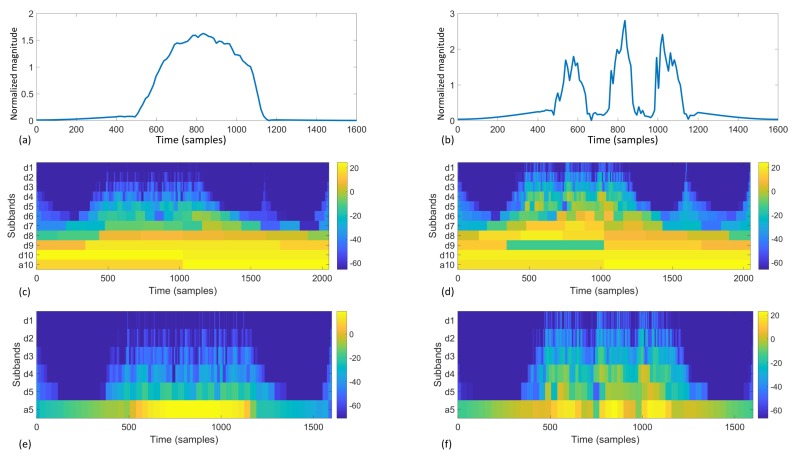
Visual illustration of wavelet transform. (**a**,**b**) are the source signal; (**c**–**f**) are scaleograms of the wavelet transform coefficients; (**c**,**e**) are the results from (**a**); (**d**,**f**) are the results from (**b**); (**c**,**d**) are the results of 10 filterbank iterations; (**e**,**f**) are 5 filterbank iterations.

We use fast wavelet transform implemented by the Large Time-Frequency Analysis Toolbox (LTFAT) [30], which follows Mallat’s basic filterbank algorithm for discrete wavelet transform [31]. Figure 8 offers an illustration and comparison of two different source signals going through 5-level and 10-level filterbanks. Essentially, each filterbank iteration calculates a vector of coefficients as results. The calculation uses a mother wavelet, which is scaled and shifted to provide frequency variance and temporal localization. In this study, we used the Daubechies 8 (db8) wavelet as the motherwavelet [32]. Other standard mother wavelets can also be used in this process; however, once chosen, the mother wavelet should not be changed because the wavelet transform will have different references. Higher iteration targets the higher frequency and finer temporal localization, which results in a longer vector of coefficients. For example, assume the sample frequency is *f*, with T=1600 samples, a five-level filterbank discrete wavelet transform as in Figure 8e,f results in wavelet coefficients as shown in Table 2. We define the highest level as *J*, therefore the coefficients are

C(Dx)=Cdj(Dx),j∈[1,J]Caj,j=J

These coefficients are unique to the specific signal, as they can be used to reconstruct the signal by the inverse wavelet transform. Each subband contains temporal localization of the corresponding frequency. Therefore their distribution information can be used as unique features.

For the last three subbands (in the example in Table 2, level 3–5, subband d3, d4, d5, a5), we calculate the $mean\left(C_{n}\left(D_{x}\right)\right),n\in \left\{aJ,dJ,d\left(J-1\right),d\left(J-2\right),d\left(J-3\right)\right\}$ as the first four wavelet features. For the lower levels $C_{d}j\left(D_{x}\right),j\in \left[1,J-4\right]$ which have significantly finer temporal granularity and bigger number of coefficients, we calculate the following features to describe the distribution information:

- $mean(C_{n}\left(D_{x}\right))$
- $max\left(C_{n}\left(D_{x}\right)\right)-min\left(C_{n}\left(D_{x}\right)\right)$
- $std(C_{n}\left(D_{x}\right))$
- $kur(C_{n}\left(D_{x}\right))$
- $skw(C_{n}\left(D_{x}\right))$

Therefore, for every sequence of frame descriptors $D_{x}$ for J=5, 14 features are calculated from the wavelet transform; for J=10, 39 features are calculated.

### 4.2. Evaluation Methods

We use the classifiers from the Matlab Classification Learner app (2017a, MathWorks, Natick, MA, USA), which enables performance comparison of various classifiers in one stop. The classifiers we evaluated are:

- Medium Tree (maximum 20 splits decision tree)
- Linear Discriminant Analysis (LDA)
- Support Vector Machine (SVM) with linear kernel
- SVM with quadratic kernel
- K-nearest neighbors (KNN) with K=10
- distance weighted KNN with K=10
- Bagged Trees (random forest bag, with decision tree learners)

For cross-validation, we consider three settings:

- Random cross-validation: the training data and testing data are from the same data set with k-fold cross-validation.
- Leave one recording out: as the data from the same experiment session may exhibit greater similarity, we use separate different sessions from the same person into training and testing data of the classifier.
- Person independent exclusive: the training data and testing data are from two groups of persons; the two groups are mutually exclusive. So that the classifier has no previous knowledge of the person being tested.

In all three settings, the testing data samples do not appear in the training data pool. The results we present are calculated from the confusion matrix after comparing the prediction with the ground truth. We use accuracy (ACC) as the comparison measure, which is the proportion of correctly classified samples in all testing data.

### 4.3. Feature Contribution Decomposition

First we investigate the contribution from different feature sets by using only the specific set of features in the machine learning process. In this step, we use the random cross-validation setting, with all the data from Group A, with 5-fold cross-validation.

#### 4.3.1. Basic Features

The basic features are defined in Section 4.1.1. We consider all the frame descriptors ($D_{x},x\in \left[1,6\right]$). The results are listed in Table 3. The average accuracy of all classifiers is 86.44%, which is well above the chance level of seven classes 14.29%. The best performed classifier is SVM with quadratic kernel with the accuracy of 91.90%.

#### 4.3.2. Wavelet Features

Different from the basic features, the amount of wavelet features depends on the filterbank iteration (*J*) of the discrete wavelet transform. Therefore, we evaluate the wavelet features by trying different filterbank iterations, as shown in Table 4. The obvious trend is that as *J* increases, the accuracy of all classifiers are increasing. On average, while J=6 and J=4, the results are inferior to the basic features, while J=8 and J=10 yields slightly better results than the basic features most of the classifiers (except for LDA and KNN). Even though there are instances that a higher *J* yields slightly lower accuracy (for example, Quadratic SVM with J=10 and J=8, it is within the random error range, because every result is from a unique randomly separated 5-fold cross validation.

#### 4.3.3. Combined Features

We combine both the basic features and wavelet features. From Table 5, all of the results are better than either basic features as in Table 3 and wavelet features from Table 4. For example, with the LDA classifier, basic features and wavelet features (J=4) yield the accuracy of 79.10% and 77.20%; while both feature sets are combined, the accuracy is improved by 4.50% to 83.60%.

We assume the application is not limited by the computational power. Therefore, after comparing the contribution of the basic features and wavelet features, we choose basic features and wavelet features (J=10) combined for the following evaluation.

#### 4.3.4. Contribution of Different Frame Descriptors

In Section 4.1, we introduce 6 *frame descriptors*. During the previous feature contribution analysis, all six frame descriptors are used. The six frame descriptors offer information from different angles: $D_{1}$ and $D_{4}$ describes the average center pressure point by the value and location; $D_{2}$ and $D_{5}$ are the maximum pressure point; $D_{3}$ is the variation of the pressure profile; $D_{6}$ measures the pressed area. Here we discuss how each frame descriptor contributes to the classification result. Table 6 shows the results of cross-validations with separate frame descriptors. The results are based on all basic features and wavelet features with J=10. Different descriptors contribute differently in combination with different classifiers. For example, $\left[D_{1},D_{4}\right]$ give less accuracy than $D_{6}$ with the KNN classifiers, but more with Quadratic SVM. Overall, the combination of all frame descriptors $\left[D_{1–6}\right]$ offer superior result than any of the individual descriptors. This means that all of the descriptors make positive contribution to the classification result.

## 5. Result and Discussion

From the previous analysis, we take all the frame descriptors, with both basic features and wavelet features (J=10), because all analysis indicate that all of the factors contribute positively to the classification result. We choose the support vector machine classifier with quadratic kernel as it offers the best accuracy.

**Figure 9:**
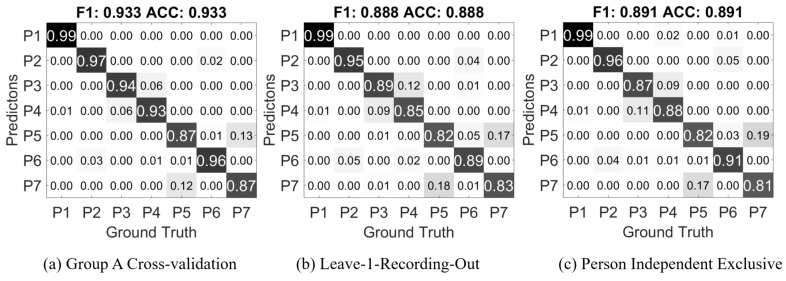
Confusion matrix of the cross-validation result on Group A dataset.

The first result in Figure 9a is from the randomly separated 5-fold cross-validation from all participants in Group A, it is essentially the confusion matrix of the corresponding result from Table 5. The values in the matrix are ratios of the current prediction in the overall ground truth of its class; on the diagonal, the values are the true positive ratio of each class. The F1 score is is calculated as the harmonic mean of the average precision and recall of all the classes; the ACC score is the accuracy, which is the average true positive rate.

As mentioned in Section 4.2, data recorded in the same session may posses greater similarity than another session from the same person. In Group A, every participant attended two recording sessions on different days. We separate these sessions as two sets, each set contains one session from each participant. One set is used as training, and the other as testing; the process is then reversed as the training and testing sets are exchanged. The confusion matrix in Figure 9b is the average of both results.

Next we evaluate how well the classifier can predict with a stranger’s data. We randomly separate the 24 participants in to 4 parties, each 6 people. Then every party is used as the testing data while the other three parties are the training data. This process is repeated 4 times so that every party is used as testing data once. The result in Figure 9c is the average confusion matrix of the four repetitions.

All three results show very well separation among all of the classes. Major miss-classification happens between *P3 Press* and *P4 Push*, *P5 Scratch* and *P7 Stroke*. *Press* and *Push* are similar actions, except *Push* has greater contact area and generally greater force; *Scratch* and *Stroke* are both repeating actions, while *Scratch* may have smaller area of contact. Overall, the average 88.8% and 89.1% accuracy in leave-1-recording-out and person independent exclusive cases are also well above the random chance level of 14.3%.

**Figure 10:**
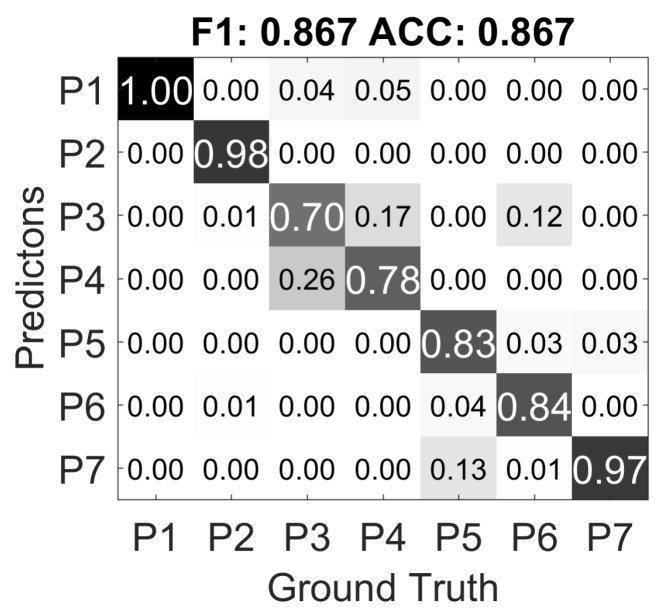
Confusion matrix of the person independent exclusive case of Group B.

As mentioned in Section 3.2, we recorded two experiment groups in different environments settings with different directing persons. The directing persons from Group A and B do not know how each other has conducted the experiment; they are only given the instructions of how to set up the sensors, and the details of the gestures as shown in Table 1. The confusion matrix of person independent exclusive validation of Group B is shown in Figure 10. Except for the miss classifications observed in Group A, more data from *P6 Pinch* are classified as *P3 Press*.

**Figure 11:**
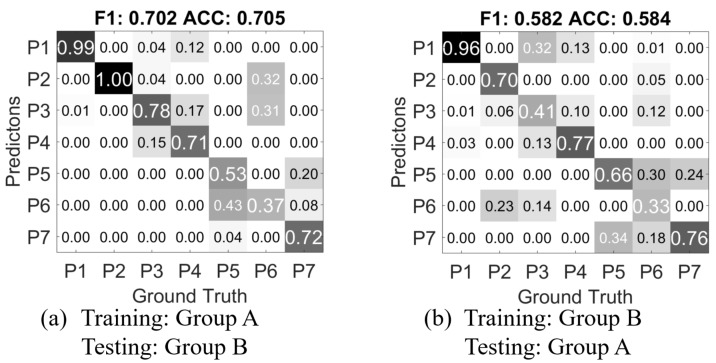
Confusion matrix of the validations using Group A and Group B as training and testing.

We then train a classifier with the feature data from Group A, and test with the data from Group B. Figure 11a shows the confusion matrix result). Compared to the confusion matrix from Figure 9c in the person independent exclusive case, *CM A-B* has near 19% drop of accuracy and F1-score. This means the mutually blinded experiment setting does decrease the recognition rate of the gesture recognition approach. Notably, 12% of *P4 Push* gestures are classified as *P1 Grab*, but most of the *P1 Grab* gestures are correctly classified. The mutual miss classifications between the pairs of *P3 Press* and *P4 Push*, *P5 scratch* and *P7 stroke*, which are observed in the cross-validation of Group A, are further increased. Most interestingly, in the Group A only cross-validation, *P6 Pinch* is clearly distinguishable from the other classes, while in *CM A-B*, it is largely miss classified into *P2 Poke* and *P3 Press*. Also gestures from *P5 Scratch* is miss-classified as *P6 Pinch*. This could be mainly caused by the mutually blind experiment setting as described in Section 3.

Figure 11b shows the result of using Group B as training, and Group A as testing (*CM B-A*). As the accuracy decreases, the classifier for *CM B-A* has only 560 samples as training data, while in *CM B-A*, the classifier is trained with 5376 samples. And also Group B contains only the data from one hand of each person, while in Group A, both hands are used for recording the data. The major miss classification caused by *P6 Pinch* also exists in *CM B-A*, which further suggests that the mutually blind setting is the underlying cause.

Overall, the comparison of *CM A-B*, *CM B-A* and the cross validations within Group A and Group B concludes that (1) a completely blinded setting regarding experiment and instruction can make a difference in recognition results; (2) more training data can improve the accuracy for person independent cases.

## 6. Conclusions and Future Work

In this work, we developed a textile robot skin prototype from tactile pressure mapping sensors and algorithms to investigate Human-Robot interface through various kinds of touch, which is still an uncharted territory. The textile touch sensing skin is soft and the feel is close to clothing materials. In a small region, it can detect different modes of touch gestures with the same skin patch through our evaluation.

Our data processing algorithm first converts the spatial pressure profiles at each sample time into frame descriptors, and calculates features from the temporal sequences of the frame descriptors. We analyzed the contribution of each frame descriptor and each feature set, with different classifiers. The overall result is that all of the frame descriptors with all of the feature sets provides the optimal classification result of 93% with a support vector machine classifier with quadratic kernel. The contribution breakdown also helps further optimizing the computational complexity. For example, with only the basic features on all the frame descriptors, the accuracy drops less than 2% from the optimal accuracy; with only $D_{1}$ and $D_{4}$ frame descriptors, the accuracy only drops 5%. This information is helpful in the future work of implementing the algorithms on the micro-controller in the sensor driving electronics.

The increased miss-classification in the exclusive person independent settings (Figure 9c and Figure 10) and the mutual blind experiment settings (Figure 11) evaluation reveals interesting aspects when it comes to strangers, which is similar to what may happen between human-human interactions: for example, someone’s normal pet on the shoulder may feel too heavy for some certain people. This opens the possibility to progressively improve tactile communication through learning or even differentiate between users of the robot through social-purposed touch sensing.

## Figures and Tables

**Table 1:** Prediction class of gestures definition.

Index	Gesture	Details
P1	grab	whole hand grabbing the dummy’s arm
P2	poke	with one finger, quick and forceful action
P3	press	with multiple finger tips, slow action
P4	push	whole hand including palm, slow action
P5	scratch	with multiple finger tips, quick repeating actions
P6	pinch	on a small area, forceful action
P7	stroke	with multiple fingers, gentle repeating actions

**Table 2:** The frequency range and coefficients of the discrete wavelet transform.

Level	Subband Index (*j*)	Frequency in fn	Frequency in Hz	Coefficients $C(D_{x})$
1	d1	fn/2–fn	25–50	$C_{d1}\left(D_{x},n\right),n\in \left[1,800\right]$
2	d2	fn/4–fn/2	12.5–25	$C_{d2}\left(D_{x},n\right),n\in \left[1,400\right]$
3	d3	fn/8–fn/2	6.25–12.5	$C_{d3}\left(D_{x},n\right),n\in \left[1,200\right]$
4	d4	fn/16–fn/8	3.125–6.25	$C_{d4}\left(D_{x},n\right),n\in \left[1,100\right]$
5	d5	fn/32–fn/16	1.5625–3.125	$C_{d5}\left(D_{x},n\right),n\in \left[1,50\right]$
5	a5	0–fn/32	0–1.5625	$C_{a5}\left(D_{x},n\right),n\in \left[1,50\right]$

**Table 3:** Classification accuracy comparison of different classifiers using basic features.

Classifier	ACC ${}^{a}$
Medium Tree	80.50%
LDA ${}^{b}$	79.10%
SVM (Linear) ${}^{c}$	90.80%
SVM (Quadratic)	91.90%
KNN (K=10) ${}^{d}$	86.20%
Weighted KNN (K=10)	87.00%
Bagged Trees	89.60%

${}^{a}$ ACC: Accuracy, the proportion of correctly classified samples in all testing data; ${}^{b}$ Linear Discriminant Analysis; ${}^{c}$ Support Vector Machine; ${}^{d}$ K-Nearest Neighbors.

**Table 4:** Classification accuracy comparison of different classifiers using wavelet features with varied levels of filterbanks.

Classifier	ACC (J=10)	ACC (J=8)	ACC (J=6)	ACC (J=4)
Medium Tree	82.10%	79.70%	78.10%	77.70%
LDA	75.00%	77.90%	74.10%	77.20%
SVM (Linear)	91.70%	91.10%	88.50%	87.10%
SVM (Quadratic)	92.30%	92.40%	89.70%	87.80%
KNN (K=10)	85.40%	84.30%	77.50%	77.00%
Weighted KNN (K=10)	86.10%	84.60%	77.80%	77.60%
Bagged Trees	91.60%	91.00%	88.00%	86.60%

**Table 5:** Classification accuracy comparison of using different classifiers with both basic and wavelet features (for wavelet features, with varying *J*).

Classifier	ACC (J=10)	ACC (J=8)	ACC (J=6)	ACC (J=4)
Medium Tree	83.80%	83.70%	83.20%	81.20%
LDA	80.20%	82.30%	81.20%	83.60%
SVM (Linear)	92.80%	92.70%	92.10%	91.70%
SVM (Quadratic)	93.30%	93.60%	92.80%	92.20%
KNN (K=10)	87.10%	86.90%	84.00%	86.00%
Weighted KNN (K=10)	87.70%	87.20%	83.90%	86.00%
Bagged Trees	92.40%	92.30%	91.50%	90.80%

**Table 6:** Comparison of the contribution of different frame descriptors, the features used are both basic and wavelet (J=10) features.

Classifier	ACC ($D_{1},D_{4}$)	ACC ($D_{2},D_{5}$)	ACC ($D_{3}$)	ACC ($D_{6}$)	ACC ($D_{1–6}$)
Medium Tree	70.90%	70.50%	71.40%	74.30%	83.80%
LDA	68.80%	67.70%	64.50%	73.00%	80.20%
SVM (Linear)	85.80%	83.40%	85.20%	85.00%	92.80%
SVM (Quadratic)	88.00%	84.60%	86.10%	85.30%	93.30%
KNN (K=10)	74.90%	73.70%	73.10%	78.80%	87.10%
Weighted KNN (K=10)	75.50%	74.70%	74.10%	79.10%	87.70%
Bagged Trees	84.90%	82.50%	83.10%	85.00%	92.40%
